# Artificial intelligence-related perceptions and E-health literacy among nursing students in Palestine: A cross-sectional study

**DOI:** 10.1371/journal.pdig.0001737

**Published:** 2026-09-25

**Authors:** Samar Thabet Jallad, Maysa Kassabry, Adel Taher Takruri

**Affiliations:** 1 Department of Nursing, Faculty of Health Professions, Al-Quds University, Jerusalem, Palestine; 2 Faculty of Nursing, Arab American University, Jenin, Palestine; 3 Grant Office, Hebron University, Hebron, Palestine; The University of Sheffield, UNITED KINGDOM OF GREAT BRITAIN AND NORTHERN IRELAND

## Abstract

Artificial intelligence (AI) and e-health literacy are increasingly relevant to nursing education; however, the relationship between nursing students’ perceptions and acceptance of AI and their perceived ability to locate, evaluate, and apply online health information remains insufficiently examined in Palestine. This cross-sectional study examined e-health literacy, AI-related technology-acceptance perceptions, Internet-use factors associated with these constructs, and whether overall AI-related perceptions were independently associated with e-health literacy among 113 third- and fourth-year nursing students. Participants completed the 8-item eHealth Literacy Scale (eHEALS) and a 28-item adapted technology-acceptance questionnaire covering seven AI-related perception domains, with negatively worded items reverse scored where required. Descriptive statistics, regrouped one-way analyses of variance, Pearson correlations with Holm adjustment for multiple testing, and multiple linear regression were performed. The mean eHEALS score was 3.53 (SD 0.59), and the overall AI-related perceptions score was 3.35 (SD 0.34). Overall AI-related perceptions were positively correlated with eHEALS (r = 0.387, Holm-adjusted p < .001). Facilitating conditions showed the largest domain-level correlation with eHEALS (r = 0.450, Holm-adjusted p < .001), whereas anxiety was not significantly associated with eHEALS. After adjustment for year of study, perceived Internet skills, and perceived importance of the Internet, overall AI-related perceptions remained independently associated with eHEALS (B = 0.643, 95% CI 0.289–0.998, p < .001; model R^2^ = 0.177). Among the regrouped Internet-use comparisons, only perceived usefulness of the Internet remained significant after Holm correction. Overall, AI-related perceptions and perceived e-health literacy were positively associated in this sample, although the cross-sectional design precludes conclusions regarding directionality or causality. Further multi-site, longitudinal, and intervention studies are warranted, and domain-level findings should be interpreted cautiously because several adapted subscales demonstrated low internal consistency.

## Author summary

Artificial intelligence is increasingly used in university learning, but students’ willingness to engage with AI and their ability to find and evaluate online health information may not develop in the same way. We surveyed 113 third- and fourth-year nursing students in Palestine using the eHealth Literacy Scale and a 28-item questionnaire covering technology-acceptance-related perceptions of AI. Students generally reported mid-range scores on both measures. After correcting for multiple statistical tests, overall AI-related perceptions remained positively associated with perceived e-health literacy, and most AI perception domains except anxiety showed positive associations. In a multivariable model that accounted for year of study, perceived Internet skills, and perceived importance of the Internet, overall AI-related perceptions remained independently associated with e-health literacy. Because the study was cross-sectional and relied on self-report, these findings do not show that AI use causes better e-health literacy. The results provide preliminary evidence for further multi-site, longitudinal, and intervention research on how nursing students’ digital health literacy and perceptions of AI develop together.

## Introduction

Digital transformation is revolutionizing the field of nursing education and health-care delivery, and consequently, there is an ever-growing demand on nurses and nursing students to work effectively and critically in technology-enabled environments [1–5]. Digital literacy in nursing is not only about having access to technology and the Internet. The students are expected not only to find and critically assess information but also to use digital learning resources effectively, adapt to new technologies, and prepare themselves for working in clinical settings where technologies like electronic information systems and decision support tools are used [2,4,5]. These expectations make digital literacy an important educational concern since using technology safely could affect how students learn, interpret evidence, communicate information, and prepare for modern-day nursing practice.

Another critical aspect of digital literacy is e-health literacy. E-health literacy can be defined as the knowledge and skills perceived to be necessary for the searching, understanding, evaluation, and utilization of health information acquired through electronic media [6]. In the case of nursing students, these competencies are pertinent to learning, evidence-based decisions, and eventually clinical practice. While the online world provides quick access to vast amounts of health information, the mere access to information does not guarantee the discernment between quality information and misinformation. Previous research conducted on nursing students found a relationship between e-health literacy and digital literacy, self-efficacy, confidence in accessing online health information, and health behaviors [7–11]. The results indicate that nursing students’ perceived skills in accessing online health information represent a more complex construct including not only their technical skills and confidence but also experience with online sources.

Artificial Intelligence (AI) is yet another aspect of the digital learning environment that is becoming more noticeable. The use of AI-enabled applications in the field of nursing education can be carried out for the purposes of information searching, simulations, adaptive learning, feedback provision, and decision support [3,12,13]. Nevertheless, the significance of AI in education should not be considered solely from the standpoint of its availability; students’ attitudes towards the technology must also be taken into account. Frameworks of technology acceptance highlight various factors like perceived usefulness, perceived ease of use, performance expectancy, facilitating conditions, attitude, and emotions [14–16]. These types of variables have also been considered in research examining digital and technology-assisted learning by nursing students [17,18], and the role of AI in nursing research has also stressed the importance of these constructs as factors that can influence behavioral intentions with respect to AI [19]. However, these variables can be described as acceptance variables as they are not indicative of the frequency of use, duration of use, or purposes for use of AI.

Thus, e-health literacy and AI-related perceptions can be viewed as different constructs, though they could have common grounds of being rooted in digital readiness. A student who is more competent in finding, analyzing, and using information on the Web related to health can be more self-confident when using new digital tools, while positive perceptions towards technology can go along with active participation in a digital environment. The existing studies indicate the relationship between digital literacy, self-efficacy, e-health literacy, and perceptions towards digital technologies in nursing students [4,8,10,11,20]. However, the relationship between e-health literacy and perceptions regarding AI is not something that can be taken for granted. It might be the case that higher e-health literacy leads to better perceptions regarding AI, better perceptions regarding technology lead to higher digital confidence, or both might be influenced by something else, such as previous training, resources available, etc. Cross-sectional analysis only allows us to spot correlations, not cause-and-effect relationships.

Such an issue is especially pertinent in cases where opportunities for access to digital materials, infrastructure, and new technologies can be different. There have been some studies of regional nursing education regarding digital health literacy and perception of AI [21]. However, there is still a lack of empirical evidence concerning Palestine. Palestinian nursing students come into contact with various online educational materials and new AI-assisted tools more often, but there is very little information on how their digital literacy perception is linked to technology-acceptance perception of AI. When all these constructs are considered together, it can lead to some early insights on the readiness of the students for their digitized environment without assuming acceptance-related perceptions to be AI use behaviors.

Accordingly, this study examined perceived e-health literacy and AI-related technology-acceptance perceptions among third- and fourth-year nursing students in Palestine. It also assessed selected Internet-use factors associated with these constructs, examined associations between seven AI-related perception domains and e-health literacy after adjustment for multiple testing, and evaluated whether the overall AI-related perceptions score remained associated with e-health literacy after adjustment for year of study, perceived Internet skills, and perceived importance of the Internet.

### Research questions

1. What are the distributions of perceived e-health literacy and AI-related technology-acceptance scores among nursing students?
2. Which selected Internet-use factors are associated with overall AI-related perceptions and e-health literacy?
3. What are the associations between the seven AI-related perception domains and e-health literacy after adjustment for multiple testing?
4. Does the overall AI-related perceptions score remain associated with e-health literacy after adjustment for year of study, perceived Internet skills, and perceived importance of the Internet?

## Methods

### Ethics statement

Ethical review documentation for this study was obtained from the Research Ethics Subcommittee of the Faculty of Health Professions, Al-Quds University (Ref. RESC/2024–42; May 23, 2024), prior to data collection, which was conducted during the Fall semester of the 2025–2026 academic year. The letter confirmed that the study proposal met the Subcommittee’s research ethics requirements and indicated that further assessment by the Central Research Ethics Committee of Al-Quds University was required. Students were informed about the purpose of the study, the voluntary nature of participation, confidentiality, and their right to decline participation without academic penalty. Completion and submission of the questionnaire were considered informed consent. Direct personal identifiers were not used in the statistical analyses.

### Study design and setting

A cross-sectional descriptive-correlational design was used [22]. The study was conducted in the Nursing Department, Faculty of Health Professions, Al-Quds University, Palestine. Data were collected during the Fall semester of the 2025–2026 academic year through an online Google Forms questionnaire from eligible third- and fourth-year nursing students enrolled in Epidemiology, Leadership and Management of Nursing, and Health Informatics courses.

### Sample and recruitment

The sampling frame comprised 150 eligible third- and fourth-year undergraduate nursing students across the participating nursing courses. All eligible students were invited to participate, constituting convenience sampling of the accessible population. Using the finite-population correction with a 95% confidence level, 5% margin of error, and expected proportion of 0.50, the minimum sample size was 109 [23]. A total of 113 students completed the questionnaire (response rate 75.3%). Thirty-seven eligible students did not participate or did not submit a completed questionnaire; individual reasons for non-completion were not available in the analytic dataset.

### Inclusion criteria

Students were eligible if they were third- or fourth-year undergraduate nursing students enrolled in one of the participating courses—Epidemiology, Leadership and Management of Nursing, or Health Informatics—during the Fall semester of the 2025–2026 academic year and agreed to participate in the study.

### Instruments

#### Part 1: Sociodemographic and Internet-use characteristics.

The demographic and Internet-use section included gender, age group, level of study, nursing course, academic average, place of residence, type of study, perceived Internet skills, perceived importance of the Internet, device most often used to access the Internet, perceived usefulness of the Internet, and frequency of Internet use for study purposes [21].

#### Part 2: eHealth Literacy Scale (eHEALS).

Perceived e-health literacy was assessed with the 8-item eHealth Literacy Scale developed by Norman and Skinner [24]. Items are rated on a 5-point response scale from strongly disagree (1) to strongly agree (5); higher mean scores indicate greater perceived e-health literacy. The questionnaire supplied for this study was administered in English. The original study reported good internal consistency, and Cronbach’s alpha in the present sample was 0.864.

#### Part 3: AI-related technology-acceptance questionnaire.

The 28-item AI questionnaire is an adapted composite of technology-acceptance-related items rather than a behavioral measure of actual AI-use frequency. The item set draws on the Technology Acceptance Model (TAM) and related acceptance constructs described by Davis [14], the UTAUT item set reported by Venkatesh et al. [15], and educational technology items used by Saadé and Kira [16]. The seven domains were performance expectancy (4 items), attitude toward using technology (4 items), facilitating conditions (4 items), affect (5 items), perceived usefulness (3 items), perceived ease of use (4 items), and anxiety (4 items). The full item list is provided as Supporting Information (S1 Text). The questionnaire was administered in English, and no translation or back-translation procedure was required.

Three experts in nursing education, nursing administration, and health policy qualitatively reviewed the adapted questionnaire for clarity, relevance, and appropriateness to the nursing education context. Following their review, minor wording modifications were made to improve contextual relevance. Generic technology-related terms such as “system,” “computer,” and “online learning system” were replaced with “AI,” and work-related expressions were adapted to reflect course, study, and academic-performance contexts. These modifications were intended to improve clarity and contextual suitability without changing the underlying meaning of the items or the prespecified domain structure. Because item-level expert-rating data were not available, a retrospective inter-rater agreement or content-validity coefficient could not be calculated.

#### Scoring and reliability.

All AI items were scored from 1 (strongly disagree) to 5 (strongly agree). To maintain a consistent direction within positive-perception domains, FC3, AFF4, AFF5, and PU1 were reverse scored. Anxiety was retained in its original direction for the anxiety subscale, so higher anxiety scores indicated greater apprehension. For the overall 28-item AI-related perceptions composite, the four anxiety items were additionally reverse scored so that a higher overall score represented more favorable AI-related perceptions and acceptance.

Internal consistency was assessed using Cronbach’s alpha (α) [25]. The overall AI-related perceptions composite had Cronbach’s α = 0.826. Domain-level coefficients were 0.680 for performance expectancy, 0.784 for attitude toward using technology, 0.302 for facilitating conditions, 0.371 for affect, 0.131 for perceived usefulness, 0.680 for perceived ease of use, and 0.704 for anxiety. Because internal consistency was low for several adapted subscales, particularly facilitating conditions, affect, and perceived usefulness, domain-specific findings were treated as exploratory and interpreted cautiously.

Although performance expectancy and perceived usefulness are conceptually related, they were retained as separate domains based on their prespecified theoretical emphasis. Performance expectancy reflects students’ expectations regarding the effects of AI on academic performance and productivity, whereas perceived usefulness reflects the perceived practical value and usefulness of AI within the course. Because their item content is conceptually close, the present study does not claim that the two domains are empirically distinct; their conceptual proximity was considered when interpreting domain-specific findings, and future psychometric work should further assess their discriminant validity.

### Data analysis

Analyses were conducted on 113 complete responses. Descriptive statistics included frequencies, percentages, means, standard deviations, and 95% confidence intervals. Equal-interval labels such as low/moderate/high were not used. Small or conceptually unstable Internet-use categories were regrouped before omnibus comparisons: Internet importance was analyzed as Important/unsure versus Very important; Internet usefulness as Low/uncertain (Not useful + Unsure), Useful, or Very useful; device as Smartphone versus Other device; and study-related Internet frequency as Less than daily, Daily, or Multiple times/day. One-way ANOVA was used for these omnibus comparisons; Levene tests did not indicate material variance heterogeneity. Pearson correlations assessed associations between the seven AI-related domains, the overall AI-related perceptions composite, and eHEALS. To control family-wise error, Holm adjustment was applied separately to the 10-omnibus group-comparison tests and the 8 Pearson correlation tests. A parsimonious multiple linear regression modeled eHEALS as the outcome, with overall AI-related perceptions as the primary explanatory variable and year of study, perceived Internet skills, and perceived Internet importance as prespecified covariates. Internet skills were entered with indicator variables and Internet importance was entered as Very important versus Important/unsure. The variance inflation factor was less than 1.9 for all predictors. Since the regression residuals had some non-normality (Shapiro-Wilk p-value = .034), HC3 robust standard errors were applied as the sensitivity-adjusted measure; the interpretation of the results was not different from the classical OLS model. The Cook’s distance indicated 7 cases with values greater than 4/n. In sensitivity analysis, removing those cases yielded the same relationship between general AI perception and eHEALS. Significance level was set to two-sided alpha = .05 after adjustment for multiple testing.

## Results

### Participants

A total of 113 students were included. Most were female (92, 81.4%) and aged 20–24 years (93, 82.3%). Third-year students accounted for 58 (51.3%) and fourth-year students for 55 (48.7%). Participants were enrolled in Epidemiology (57, 50.4%), Leadership and Management of Nursing (34, 30.1%), or Health Informatics (22, 19.5%). Most reported good Internet skills (70, 61.9%) and most often accessed the Internet by smartphone (79, 69.9%). Full characteristics are shown in Table 1.

**Table 1:** Demographic and Internet-use characteristics of participants (n = 113).

Variable	Category	n (%)
Gender	Male	21 (18.6)
	Female	92 (81.4)
Age (years)	<20	9 (8.0)
	20-24	93 (82.3)
	25-29	8 (7.1)
	>29	3 (2.7)
Level of study	Third year	58 (51.3)
	Fourth year	55 (48.7)
Nursing course	Epidemiology	57 (50.4)
	Leadership and Management of Nursing	34 (30.1)
	Health Informatics	22 (19.5)
Academic average	≤69%	2 (1.8)
	70-79%	51 (45.1)
	80-89%	56 (49.6)
	90-100%	4 (3.5)
Place of residence	City	49 (43.4)
	Village	57 (50.4)
	Camp	7 (6.2)
Type of study	Regular	94 (83.2)
	Upgrading	19 (16.8)
Perceived Internet skills	Average	22 (19.5)
	Good	70 (61.9)
	Very good	21 (18.6)
Perceived importance of Internet	Unsure	9 (8.0)
	Important	69 (61.1)
	Very important	35 (31.0)
Device most used	Smartphone	79 (69.9)
	Laptop	21 (18.6)
	Tablet	7 (6.2)
	Computer	6 (5.3)
Perceived usefulness of Internet	Not useful	2 (1.8)
	Unsure	15 (13.3)
	Useful	74 (65.5)
	Very useful	22 (19.5)
Internet use for study	Sometimes in a month	4 (3.5)
	Once a week	16 (14.2)
	Every day	57 (50.4)
	1-2 times/day	16 (14.2)
	>3 times/day	20 (17.7)

### Descriptive scores and reliability

The mean eHEALS score was 3.53 (SD 0.59, 95% CI 3.42-3.64). The highest eHEALS item mean was for using online health information to help oneself (M = 3.65), whereas the lowest was for knowing what health resources are available online (M = 3.41). The overall positive-direction AI-related perceptions score was 3.35 (SD 0.34, 95% CI 3.29-3.41). Performance expectancy and perceived ease of use had the largest domain means, while the anxiety subscale had a mean of 3.09 (SD 0.63), where higher values indicate greater anxiety. Table 2 reports means, confidence intervals, and internal consistency coefficients without categorical labels.

**Table 2:** Descriptive statistics and internal consistency for eHEALS and AI-related perception scores (n = 113).

Code	Scale/domain/item	Mean	SD	95% CI	Cronbach α
E1	I know what health resources are available on the Internet	3.41	0.86	3.25-3.57	
E2	I know where to find helpful health resources on the Internet.	3.54	0.85	3.38–3.70	
E3	I know how to find helpful health resources on the Internet	3.56	0.82	3.40–3.71	
E4	I know how to use the Internet to answer my questions about health	3.64	0.82	3.48–3.79	
E5	I know how to use the health information I find on the Internet to help me.	3.65	0.75	3.51–3.80	
E6	I have the skills I need to evaluate the health resources I find on the Internet	3.55	0.82	3.40–3.70	
E7	I can tell high quality health resources from low quality health resources on the Internet	3.45	0.80	3.30–3.60	
E8	I feel confident in using information from the Internet to make health decisions	3.42	0.81	3.27–3.58	
	Total eHEALS	3.53	0.59	3.42–3.64	0.864
	Performance expectancy	3.60	0.54	3.49–3.70	0.680
	Attitude toward using technology	3.50	0.62	3.38–3.61	0.784
	Facilitating conditions	3.25	0.46	3.17–3.34	0.302
	Affect	3.30	0.46	3.21–3.38	0.371
	Perceived usefulness	3.35	0.51	3.25–3.44	0.131
	Perceived ease of use	3.56	0.51	3.47–3.66	0.680
	Anxiety	3.09	0.63	2.97–3.20	0.704
	Overall AI-related perceptions	3.35	0.34	3.29–3.41	0.826

Note. FC3, AFF4, AFF5, and PU1 were reverse scored for their positive-perception domain scores. Anxiety is shown in its original direction (higher = greater anxiety); anxiety items were reverse scored only when calculating the overall AI-related perceptions composite. No low/moderate/high categorical labels were applied.

### Internet-use factors

After regrouping small or conceptually uncertain categories, 10 omnibus comparisons were examined and Holm correction was applied across this family of tests. Perceived usefulness of the Internet was associated with overall AI-related perceptions, F(2,110) = 5.71, unadjusted p = .004, Holm-adjusted p = .044, eta-squared = .094. Tukey comparisons indicated that students who rated the Internet as very useful had higher AI-related perception scores than the low/uncertain group (mean difference = 0.35, adjusted p = .003); the remaining pairwise comparisons were not statistically significant. No other Internet-use factor remained significant after Holm correction, including perceived importance of the Internet. Table 3 presents the complete results.

**Table 3:** Regrouped Internet-use factors associated with overall AI-related perceptions and eHEALS (n = 113).

Factor	Outcome	Omnibus test	Raw p	Holm p	η²
Perceived Internet skills	Overall AI-related perceptions	F(2,110) = 1.63	.200	.800	0.029
Perceived Internet skills	eHEALS	F(2,110) = 1.21	.301	.800	0.022
Perceived importance of Internet	Overall AI-related perceptions	F(1,111) = 3.26	.074	.516	0.029
Perceived importance of Internet	eHEALS	F(1,111) = 3.78	.054	.436	0.033
Device most used	Overall AI-related perceptions	F(1,111) = 1.65	.202	.800	0.015
Device most used	eHEALS	F(1,111) = 2.44	.121	.604	0.022
Perceived usefulness of Internet	Overall AI-related perceptions	F(2,110) = 5.71	.004	.044	0.094
Perceived usefulness of Internet	eHEALS	F(2,110) = 1.29	.279	.800	0.023
Internet use for study	Overall AI-related perceptions	F(2,110) = 2.51	.086	.517	0.044
Internet use for study	eHEALS	F(2,110) = 3.27	.042	.377	0.056

Note. Holm adjustment was applied across the 10 omnibus tests in this table. Regrouping was performed before testing to avoid very small or conceptually unstable cells. Only perceived usefulness of the Internet for overall AI-related perceptions remained statistically significant after Holm correction.

### Correlations

Holm-adjusted Pearson correlations are shown in Table 4. Overall, AI-related perceptions were positively associated with eHEALS (r = 0.387, 95% CI 0.218-0.534, Holm-adjusted p < .001). Facilitating conditions showed the largest domain-level association (r = 0.450), followed by perceived ease of use (r = 0.364). Performance expectancy, attitude, affect, and perceived usefulness also showed positive associations that remained statistically significant after Holm adjustment. Anxiety was not associated with eHEALS (r = 0.052, Holm-adjusted p = 0.585). Given the low alpha coefficients for several adapted domains, these domain-level correlations should be interpreted cautiously.

**Table 4:** Pearson correlations of AI-related perception domains with eHEALS (n = 113).

AI-related measure	r	95% CI	Raw p	Holm p
Performance expectancy	0.263	0.082 to 0.427	.005	.020
Attitude toward using technology	0.297	0.119 to 0.457	.001	.007
Facilitating conditions	0.450	0.290 to 0.586	<.001	<.001
Affect	0.234	0.051 to 0.401	.013	.025
Perceived usefulness	0.254	0.072 to 0.419	.007	.020
Perceived ease of use	0.364	0.192 to 0.514	<.001	<.001
Anxiety	0.052	-0.134 to 0.234	.585	.585
Overall AI-related perceptions	0.387	0.218 to 0.534	<.001	<.001

Note. Holm adjustment was applied across the eight correlations. Domain-level findings should be interpreted in light of the internal consistency coefficients reported in Table 2.

### Multivariable analysis

The adjusted regression model was statistically significant (R^2^ = 0.177, adjusted R^2^ = 0.138; robust F(5,107) = 4.19, p = 0.002). Overall, AI-related perceptions remained independently associated with eHEALS after adjustment for year of study, perceived Internet skills, and perceived Internet importance (B = 0.643, HC3 SE = 0.179, standardized beta = 0.371, 95% CI 0.289-0.998, p < .001). None of the covariates was statistically significant in the full-sample model. VIF values ranged from 1.05 to 1.87, indicating no problematic multicollinearity (Table 5). Excluding the 7 observations above the Cook’s-distance 4/n screening threshold did not weaken the primary association (B = 0.714, HC3 95% CI 0.438-0.990, p < .001).

**Table 5:** Multiple linear regression for eHEALS (n = 113).

Variable	B	HC3 SE	β	95% CI	p	VIF
Overall AI-related perceptions	0.643	0.179	0.371	0.289 to 0.998	<.001	1.05
Fourth year (vs third year)	−0.047	0.111	−0.040	−0.266 to 0.172	.670	1.10
Internet skills: Good (vs Average)	−0.093	0.167	−0.078	−0.425 to 0.238	.578	1.85
Internet skills: Very good (vs Average)	0.064	0.203	0.043	−0.338 to 0.466	.752	1.87
Internet importance: Very important (vs Important/unsure)	0.146	0.117	0.115	−0.087 to 0.378	.217	1.12

Note. Outcome = mean eHEALS score. Model R^2^ = 0.177; adjusted R^2^ = 0.138; robust F(5,107) = 4.19, p = 0.002. HC3 robust standard errors are reported. β is standardized coefficient.

## Discussion

This study examined perceived e-health literacy and AI-related technology-acceptance perceptions among nursing students using multiplicity-adjusted analyses and a parsimonious multivariable model. Overall AI-related perceptions were positively associated with eHEALS (r = 0.387, Holm-adjusted p < .001), and remained significant after adjustment for year of study, perceived Internet skills, and perceived importance of the Internet (B = 0.643, 95% CI 0.289-0.998, p < .001). Facilitating conditions showed the largest domain-level correlation, followed by perceived ease of use, whereas anxiety was not associated with eHEALS. These findings indicate co-occurrence between the constructs but do not establish direction or causality.

Participants generally perceived themselves as capable of finding and using online health information, although confidence varied across tasks. Scores were higher for using online health information to help oneself and answering health-related questions, whereas the lowest item mean concerned knowing what health resources were available online. E-health literacy requires not only access but also resource identification, credibility appraisal, and appropriate application [6]. Previous nursing-student research has linked e-health literacy with digital literacy, self-efficacy, confidence in managing online health information, and health-related behaviors [7–11]. The present pattern suggests that practical use of online information may be stronger than confidence in navigating resource quality and availability. Because eHEALS is self-reported, it reflects perceived rather than objectively demonstrated competence.

Performance expectancy and perceived ease of use had the largest AI-related mean scores, indicating that students tended to view AI as potentially beneficial for academic performance and relatively manageable to use. Technology-acceptance frameworks identify performance benefits, usefulness, and ease of use as central components of favorable technology perceptions [14,15], and related constructs have been applied in nursing education [17,18]. AI-focused nursing research has also emphasized attitudes, anxiety, self-efficacy, and ethical awareness [19]. The questionnaire did not measure behavioral engagement; therefore, higher scores represent favorable expectations or perceptions, not evidence of frequent, effective, or safe AI use.

The revised regression supports the primary finding through demonstrating that the relationship between general perception of AI and eHEALS cannot be attributed to the year of study, perception of Internet skills, and perception of importance of the Internet. The two constructs can have common dimensions of the digital readiness: students who perceive themselves to be better able to assess the online health information can also be more comfortable with emerging technologies. Previous studies describe relationships among digital literacy, self-efficacy, e-health literacy, and technology-related attitudes in nursing students [4,10,11,20]. Residual confounding remains possible, however; prior AI exposure, formal training, faculty support, institutional resources, socioeconomic conditions, or general digital self-efficacy could influence both constructs.

At the domain level, facilitating conditions had the strongest correlation with eHEALS, followed by perceived ease of use. Students who perceive adequate resources, knowledge, and support for technology use may also report greater confidence using electronic health information. This is consistent with UTAUT-based work emphasizing enabling resources [15,26] and nursing-education literature showing that digital competence depends on both individual capability and the learning environment [2,5]. However, facilitating conditions had low internal consistency in this sample (alpha = 0.302), so the magnitude of this association should be considered exploratory rather than definitive.

Performance expectancy, attitude, affect, and perceived usefulness also remained positively associated with eHEALS after Holm adjustment, while anxiety did not. These associations were generally modest, and affect and perceived usefulness require particular caution because those subscales also showed low internal consistency. Performance expectancy and perceived usefulness were retained separately because the former emphasized expected academic performance and productivity whereas the latter emphasized practical course value. Their conceptual overlap means empirical distinctiveness cannot be assumed; future factor-analytic and discriminant-validity assessment is warranted [14–16]. The absence of an anxiety association suggests that AI apprehension may not simply be the inverse of digital-health confidence and may depend on tool-specific experiences or perceived risks [19].

After regrouping small or uncertain categories and applying Holm correction across 10 omnibus comparisons, only perceived usefulness of the Internet remained associated with overall AI-related perceptions. Perceived Internet skills, perceived importance, device type, and study-related Internet-use frequency did not remain significant. This distinction matters because frequency of access is not equivalent to the evaluative competencies represented by e-health literacy [6,8,10,11]. Perceiving the Internet as useful may reflect a broader positive orientation toward digital resources that coexists with favorable AI-related perceptions, but the direction of this relationship cannot be determined.

This study adds preliminary evidence from the Palestinian nursing-education context, where research examining e-health literacy together with AI-related perceptions remains limited. Students’ experiences may vary according to infrastructure, curriculum exposure, faculty readiness, and opportunities to use digital resources [3–5,21]. Findings should therefore be interpreted within the single-university context and alongside the limited reliability of several adapted subscales. Multi-site and longitudinal studies are needed to test reproducibility and temporal ordering. Future work should directly measure AI-use frequency, duration, purposes, and specific platforms, and strengthen psychometric validation of the adapted domains before domain-level findings are used for high-stakes educational decisions.

### Limitations

However, there are some limitations associated with this research study. Firstly, it is associated with a convenience sampling strategy from one particular institution, thereby reducing its generalizability. Secondly, the use of a cross-sectional study design does not allow making any causal inferences. Thirdly, both eHEALS and the measures related to AI were self-reported measures and could therefore be affected by social desirability and/or response bias, since no measures of frequency or length of actual usage or the particular tools used in this process have been assessed. Fourth, 37 of 150 eligible students did not provide complete responses, and information was not available to compare respondents with nonrespondents; therefore, non-response bias cannot be excluded. Fifth, there is considerable variation in the interfaces, functionalities, and educational affordances provided by different AI platforms; however, the questionnaire was unable to distinguish between platforms such as ChatGPT, Claude, or Gemini. Sixth, many adapted AI scales had low internal consistency reliability in this study, specifically for facilitative conditions, affective factors, and perceived usefulness. Lastly, the model used few covariates.

### Implications and research priorities

The present findings identify questions for future nursing-education research rather than establishing a specific curricular intervention. Replication across multiple institutions should test whether the association between e-health literacy and AI-related perceptions persists in different student populations and resource settings. Longitudinal studies can clarify temporal ordering, and small controlled educational trials can evaluate whether targeted training in digital health appraisal or responsible AI use changes either construct. Future measurement work should also distinguish actual AI-use behavior from acceptance-related perceptions and should validate the adapted domains before they are used for high-stakes comparisons. Future research should also record the specific AI platforms used by nursing students and examine whether differences in platform characteristics are associated with AI-related perceptions and e-health literacy outcomes.

## Conclusion

Among 113 nursing students, overall AI-related technology-acceptance perceptions were positively associated with perceived e-health literacy, and this association remained in a parsimonious adjusted regression model. Facilitating conditions and perceived ease of use showed the largest domain-level correlations, while anxiety was not associated with eHEALS. Because the study was cross-sectional, based on self-report, and used several adapted subscales with limited reliability, the results should be interpreted as preliminary associations rather than evidence that AI use causes improved e-health literacy. Multi-site validation, longitudinal research, and intervention studies are warranted.

## Supporting information

S1 Text Complete study questionnaire, including sociodemographic and Internet-use items, the 28 AI-related technology-acceptance items, and the 8-item eHEALS. (DOCX)

S1 Data De-identified minimal dataset underlying the analyses reported in this study. (CSV)
