# Supplementary material for: Artificial intelligence-related perceptions and E-health literacy among nursing students in Palestine: A cross-sectional study
Source: PLOS Digit Health. 2026 Sep 25;5(9):e0001737. doi: 10.1371/journal.pdig.0001737 (PMC13614630; doi:10.1371/journal.pdig.0001737)
Supplement: Supplementary file 1 [file pdig.0001737.s001.docx]

Study Questionnaire

**e-Health Literacy and Artificial Intelligence–Related Perceptions Among Nursing Students**

**Instructions:** Please select the response that best represents you. For Sections B and C, use the 5-point Likert scale: Strongly Disagree, Disagree, Neutral, Agree, or Strongly Agree.

# Section A. Sociodemographic, Academic, and Internet-Use Characteristics

Please select one response for each item unless otherwise indicated.

| **1. Gender** | ☐ Female ☐ Male |
| --- | --- |
| **2. Age (years)** | ☐ <20 ☐ 20–24 ☐ 25–29 ☐ >29 |
| **3. Level of study** | ☐ 3rd year ☐ 4th year |
| **4. Nursing course** | ☐ Epidemiology ☐ Leadership and Management of Nursing ☐ Health Informatics |
| **5. Academic average** | ☐ ≤69% ☐ 70–79% ☐ 80–89% ☐ 90–100% |
| **6. Place of residence** | ☐ City ☐ Village ☐ Camp |
| **7. Type of study** | ☐ Regular ☐ Upgrading |
| **8. Perceived level of Internet skills** | ☐ Average ☐ Good ☐ Very good |
| **9. Perceived importance of the Internet** | ☐ Unsure ☐ Important ☐ Very important |
| **10. Device most used to access the Internet** | ☐ Smartphone ☐ Laptop ☐ Tablet ☐ Computer |
| **11. Perceived usefulness of the Internet** | ☐ Not useful ☐ Unsure ☐ Useful ☐ Very useful |
| **12. Frequency of using the Internet for study purposes** | ☐ Sometimes in a month ☐ Once a week ☐ Every day ☐ Fewer than 3 times a day ☐ More than 3 times a day |

# Section B. Artificial Intelligence–Related Perceptions / Technology Acceptance Items (28 items)

**Framework/reference:** The study instrument was described as adapted from the Technology Acceptance Model (TAM). Davis (1989) is the foundational TAM reference cited for perceived usefulness, perceived ease of use, and technology acceptance. The 28-item set used in this study is organized into seven domains as represented in the study dataset.

| **Domain** | **Item codes** | **Number of items** |
| --- | --- | --- |
| Performance Expectancy | PE1–PE4 | 4 |
| Attitude Toward Using Technology | ATT1–ATT4 | 4 |
| Facilitating Conditions | FC1–FC4 | 4 |
| Affect | AFF1–AFF5 | 5 |
| Perceived Usefulness | PU1–PU3 | 3 |
| Perceived Ease of Use | PEOU1–PEOU4 | 4 |
| Anxiety | ANX1–ANX4 | 4 |

## Performance Expectancy (4 items)

| **Code** | **Item** | **Strongly Disagree** | **Disagree** | **Neutral** | **Agree** | **Strongly Agree** |
| --- | --- | --- | --- | --- | --- | --- |
| PE1 | I would find AI useful in my course. | ☐ | ☐ | ☐ | ☐ | ☐ |
| PE2 | Using AI enables me to accomplish study more quickly. | ☐ | ☐ | ☐ | ☐ | ☐ |
| PE3 | Using AI increases my productivity. | ☐ | ☐ | ☐ | ☐ | ☐ |
| PE4 | If I use AI, I will increase my chances of getting a raise of my average. | ☐ | ☐ | ☐ | ☐ | ☐ |

## Attitude Toward Using Technology (4 items)

| **Code** | **Item** | **Strongly Disagree** | **Disagree** | **Neutral** | **Agree** | **Strongly Agree** |
| --- | --- | --- | --- | --- | --- | --- |
| ATT1 | Using AI is a bad/good idea. | ☐ | ☐ | ☐ | ☐ | ☐ |
| ATT2 | AI makes study more interesting. | ☐ | ☐ | ☐ | ☐ | ☐ |
| ATT3 | Studying with AI is fun. | ☐ | ☐ | ☐ | ☐ | ☐ |
| ATT4 | I like studying with AI. | ☐ | ☐ | ☐ | ☐ | ☐ |

## Facilitating Conditions (4 items)

| **Code** | **Item** | **Strongly Disagree** | **Disagree** | **Neutral** | **Agree** | **Strongly Agree** |
| --- | --- | --- | --- | --- | --- | --- |
| FC1 | I have the resources necessary to use AI. | ☐ | ☐ | ☐ | ☐ | ☐ |
| FC2 | I have the knowledge necessary to use AI. | ☐ | ☐ | ☐ | ☐ | ☐ |
| FC3 | AI is not compatible with other technology I use. | ☐ | ☐ | ☐ | ☐ | ☐ |
| FC4 | A specific person (or group) is available for assistance with AI difficulties. | ☐ | ☐ | ☐ | ☐ | ☐ |

## Affect (5 items)

| **Code** | **Item** | **Strongly Disagree** | **Disagree** | **Neutral** | **Agree** | **Strongly Agree** |
| --- | --- | --- | --- | --- | --- | --- |
| AFF1 | I like working with AI. | ☐ | ☐ | ☐ | ☐ | ☐ |
| AFF2 | I look forward to those aspects of my course work that require me to use AI. | ☐ | ☐ | ☐ | ☐ | ☐ |
| AFF3 | Once I start working on AI, I find it hard to stop. | ☐ | ☐ | ☐ | ☐ | ☐ |
| AFF4 | Using AI is frustrating for me. | ☐ | ☐ | ☐ | ☐ | ☐ |
| AFF5 | I get bored quickly when working on AI. | ☐ | ☐ | ☐ | ☐ | ☐ |

## Perceived Usefulness (3 items)

| **Code** | **Item** | **Strongly Disagree** | **Disagree** | **Neutral** | **Agree** | **Strongly Agree** |
| --- | --- | --- | --- | --- | --- | --- |
| PU1 | Using AI would reduce my ability to perform well in the course? | ☐ | ☐ | ☐ | ☐ | ☐ |
| PU2 | I think that an AI should be part of each and every course in the university. | ☐ | ☐ | ☐ | ☐ | ☐ |
| PU3 | Using the AI in the course would enhance my performance in the course. | ☐ | ☐ | ☐ | ☐ | ☐ |

## Perceived Ease of Use (4 items)

| **Code** | **Item** | **Strongly Disagree** | **Disagree** | **Neutral** | **Agree** | **Strongly Agree** |
| --- | --- | --- | --- | --- | --- | --- |
| PEOU1 | I think that learning to navigate AI will be easy for me. | ☐ | ☐ | ☐ | ☐ | ☐ |
| PEOU2 | I think that I will find it easy to get AI to do what I want them to do. | ☐ | ☐ | ☐ | ☐ | ☐ |
| PEOU3 | I think that it would be easy for me to become skillful at using AI. | ☐ | ☐ | ☐ | ☐ | ☐ |
| PEOU4 | I think that I will find the AI easy to use. | ☐ | ☐ | ☐ | ☐ | ☐ |

## Anxiety (4 items)

| **Code** | **Item** | **Strongly Disagree** | **Disagree** | **Neutral** | **Agree** | **Strongly Agree** |
| --- | --- | --- | --- | --- | --- | --- |
| ANX1 | I feel apprehensive about using AI. | ☐ | ☐ | ☐ | ☐ | ☐ |
| ANX2 | It scares me to think that I could lose a lot of information using AI by hitting the wrong action. | ☐ | ☐ | ☐ | ☐ | ☐ |
| ANX3 | I hesitate to use AI for fear of making mistakes I cannot correct. | ☐ | ☐ | ☐ | ☐ | ☐ |
| ANX4 | AI is somewhat intimidating to me. | ☐ | ☐ | ☐ | ☐ | ☐ |

# Section C. e-Health Literacy Scale (eHEALS) (8 items)

**Source:** Norman and Skinner (2006). The eHEALS is an 8-item measure of perceived eHealth literacy skills.

| **Code** | **Item** | **Strongly Disagree** | **Disagree** | **Neutral** | **Agree** | **Strongly Agree** |
| --- | --- | --- | --- | --- | --- | --- |
| eHEALS1 | I know what health resources are available on the Internet. | ☐ | ☐ | ☐ | ☐ | ☐ |
| eHEALS2 | I know where to find helpful health resources on the Internet. | ☐ | ☐ | ☐ | ☐ | ☐ |
| eHEALS3 | I know how to find helpful health resources on the Internet. | ☐ | ☐ | ☐ | ☐ | ☐ |
| eHEALS4 | I know how to use the Internet to answer my questions about health. | ☐ | ☐ | ☐ | ☐ | ☐ |
| eHEALS5 | I know how to use the health information I find on the Internet to help me. | ☐ | ☐ | ☐ | ☐ | ☐ |
| eHEALS6 | I have the skills I need to evaluate the health resources I find on the Internet. | ☐ | ☐ | ☐ | ☐ | ☐ |
| eHEALS7 | I can tell high quality health resources from low quality health resources on the Internet. | ☐ | ☐ | ☐ | ☐ | ☐ |
| eHEALS8 | I feel confident in using information from the Internet to make health decisions. | ☐ | ☐ | ☐ | ☐ | ☐ |

# References

Davis, F. D. (1989). Perceived usefulness, perceived ease of use, and user acceptance of information technology. *MIS Quarterly, 13*(3), 319–340. <https://doi.org/10.2307/249008>

Norman, C. D., & Skinner, H. A. (2006). eHEALS: The eHealth Literacy Scale. *Journal of Medical Internet Research, 8*(4), e27. <https://doi.org/10.2196/jmir.8.4.e27>
